# Noninvasive Thermographic Photographing as an Assessment of the State of Discomfort in a Dog Receiving Radiation Therapy

**DOI:** 10.3390/ani11092496

**Published:** 2021-08-25

**Authors:** Kaori Saeki, Kenji Kutara, Eri Iwata, Masahiro Miyabe, Yuki Shimizu, Yuko Wada, Akihiro Ohnishi, Akira Matsuda, Takako Shimokawa Miyama, Taketoshi Asanuma

**Affiliations:** 1 Faculty of Veterinary Medicine, Okayama University of Science, Imabari 794-8555, Japan; k-saeki@vet.ous.ac.jp (K.S.); k-kutara@vet.ous.ac.jp (K.K.); m-miyabe@vet.ous.ac.jp (M.M.); a-oonishi@vet.ous.ac.jp (A.O.); a-matsuda@vet.ous.ac.jp (A.M.); t-shimokawa@vet.ous.ac.jp (T.S.M.); t-asanuma@vet.ous.ac.jp (T.A.); 2 Veterinary Medical Teaching Hospital, Okayama University of Science, Imabari 794-8555, Japan; y-shimizu@vet.ous.ac.jp (Y.S.); y-wada@vet.ous.ac.jp (Y.W.)

**Keywords:** nasal tumors, radiation therapy, thermography, veterinary nursing

## Abstract

**Simple Summary:**

In humans, radiation induces dilation of the capillaries and inflammatory reactions, which raise skin temperature; and thermography is used to detect abnormalities after radiation therapy. However, in the field of veterinary nursing, there are no reports objectively evaluating the condition of dogs after radiation therapy using thermography. Therefore, we aimed to investigate the nasal irradiation temperature, behavioral changes, and post-irradiation pain scores in a dog receiving radiation therapy for intranasal tumors. There were no differences in behavior before and after irradiation, and pain scores were low, but the nasal planum temperature increased significantly after irradiation. Since there are individual differences in stress-related behaviors, such as pain and discomfort, assessing a dog’s pain using only subjective assessment methods, such as appearance and behavioral evaluation, is limited. In this study, we used thermography to understand changes in conditions that could not be detected by routine monitoring alone. This method is non-invasive, objective, and indispensable for proposing appropriate care.

**Abstract:**

In humans, radiation induces dilation of capillaries and inflammatory reactions to raise skin temperature. Thermography is used to detect abnormalities after radiation therapy (RT). However, in veterinary nursing, objective evaluation of the condition of dogs after RT using thermography has not been reported. We investigated the nasal irradiation temperature, behavioral changes, and post-irradiation pain scores in a dog receiving RT for intranasal tumors. The temperature of the nasal planum gradually increased after irradiation, reaching a significantly higher value at 120–240 min. The highest temperature was 42.3 °C and the average temperature increased by 4.4 °C. Behavioral analysis pre- and post-RT did not vary significantly. Post-RT pain levels evaluated by the pain scale ranged from 0 to 1 throughout. No veterinary treatment was provided. In humans, increased skin temperature after radiation causes psychological stress, i.e., pain and discomfort, but no such behavioral changes were observed in this case. Given individual differences in stress-related behaviors, such as pain and discomfort, assessing a dog’s painfulness using only subjective methods, such as appearance and behavioral evaluation, is limited. We used thermography to assess changes in conditions not detectable by routine monitoring alone. This method is non-invasive, objective, and indispensable for providing appropriate care.

## 1. Introduction

Thermography is a technique for measuring the temperatures of objects remotely and representing said temperature results through images. It is a non-invasive and safe method for detecting changes in surface temperature [1,2,3]. In dogs, thermography is primarily used to identify inflammation, determine stress, or assess diseases that have thermal effects [4,5].

Radiation therapy (RT) is a painless local therapy with fewer systemic side effects than chemotherapy. However, there are concerns regarding side effects due to the effects on normal tissues at the irradiation sites [6,7,8]. In humans, radiation has been reported to increase skin temperature by inducing capillary dilation and inflammatory reactions; skin temperature changes have been actively studied using thermography as a predictor for early detection of abnormalities after RT [9,10,11]. In addition, changes in skin temperature after RT have been reported to be more beneficial as an assessment tool than visual findings when correlated with subjective data, such as pain and discomfort [12].

Among canine nasal tumors, more than 80% are malignant and entail poor long-term prognoses [13,14]. Most dogs present to the clinic with the chief complaint of hemorrhagic or mucopurulent nasal discharge [15,16,17,18]. RT is the recommended treatment and has been reported to increase the median survival time by approximately three times compared to surgical treatment or chemotherapy alone [13,19,20,21].

Patient complaints, such as pain and discomfort, are essential information for detecting abnormalities in the irradiated and surrounding areas. However, in animals, which are unable to express their discomfort verbally, the detection of symptoms is often delayed. Therefore, early detection of abnormalities through objective observation is required. However, there are no reports of objective evaluation of the condition of animals after RT using thermography in the veterinary nursing field. Therefore, this report focuses on the evaluation of a dog with intranasal tumors receiving RT, using thermography of the nose, which is a site where the body temperature of animals can be relatively accurately read [4].

## 2. Materials and Methods

### 2.1. Case Presentation

**Figure 1:**
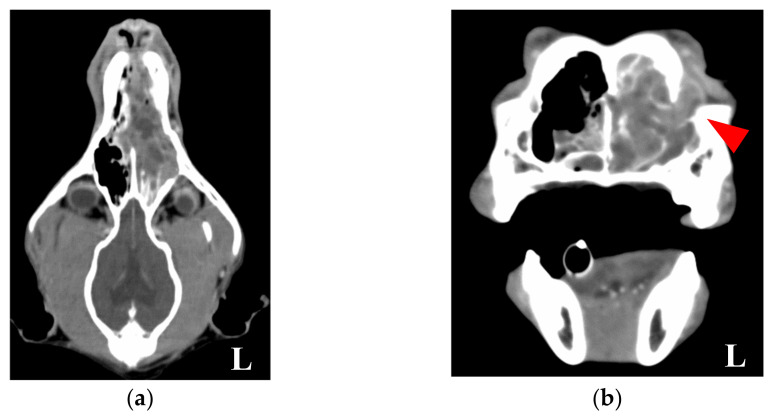
A contrast computed tomography image of the head. (**a**) Horizontal image. (**b**) Transverse image. A large, mild contrast-enhancing soft-tissue attenuating mass was present in the left nasal cavity. This mass extended caudally to the level of the cribriform plate. Partial lysis of the nasal septum was observed, and the left nasal mass invaded the right nasal cavity. Lysis of the lacrimal bone (**red arrowhead**) and mild invasion of the mass in the left orbit were observed. L: left.

A 10 year old, 17.7 kg, spayed female Shikoku Inu (Japanese native breed) was referred to the Okayama University of Science Veterinary Teaching Hospital for a 1-month history of intermittent left-sided epistaxis. Physical examination was unremarkable, except for a small amount of left-sided epistaxis. A complete blood count revealed mild anemia, but the chemistry and coagulation panels were within normal limits. Thoracic radiographic findings were unremarkable, except for a cave-in to the ventral sternum from the right-lateral view. Whole-body computed tomography (CT) was performed using a 16-slice multidetector CT scanner (Aquilion Lightning, Canon Medical Systems, Otawara, Japan) under general anesthesia. Transverse images of the head and chest were acquired before and after intravenous administration of 2 mL/kg iopamidol (Oypalomin 300, Fuji Pharma Co., Ltd., Tokyo, Japan). There was a large, expansile, mild contrast-enhancing soft-tissue attenuating mass occupying the left nasal cavity (Figure 1a). This mass extended caudally to the level of the cribriform plate and ventrally within the ventral nasal meatus to the level of the choana. Partial lysis of the nasal septum was observed, and the left nasal mass invaded the right nasal cavity. In addition, lysis of the lacrimal bone and mild invasion of the mass in the left orbit were observed (Figure 1b).

There was no evidence of metastasis on the chest CT images. A histopathological examination was performed using a straw biopsy of the occupied nasal cavity lesion. Histopathological evaluation was performed, and chondrosarcoma was diagnosed.

**Figure 2:**
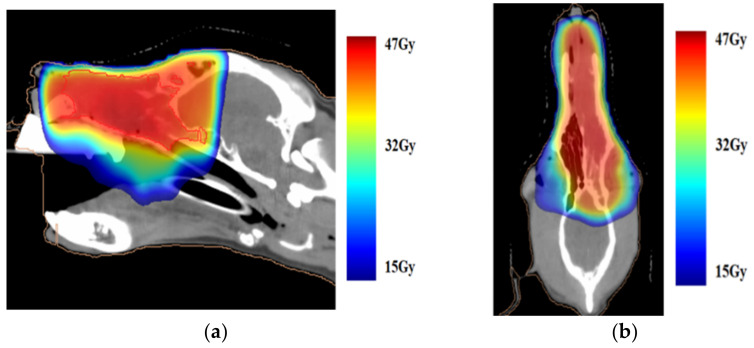
(**a**) Sagittal and (**b**) horizontal images prior to radiation therapy overlayed with the dose color wash. The whole nasal cavity is reserved for a total dose of 47 Gy.

Two weeks after the initial CT scan, based on the imaging and histopathological examination results, the dog was treated with RT. RT was performed using a linear accelerator (Elekta Synergy, Canon Medical Systems, Otawara, Japan) for a total of 15 sessions over 4 weeks with a total dose of 47.6 Gy under general anesthesia. Pre- and post-contrast CT images were acquired and used for RT planning. The gross tumor volume (GTV) included all of the grossly visible abnormal tissue. A uniform 5 mm expansion around the GTV is defined as the planned target volume (Figure 2).

**Figure 3:**
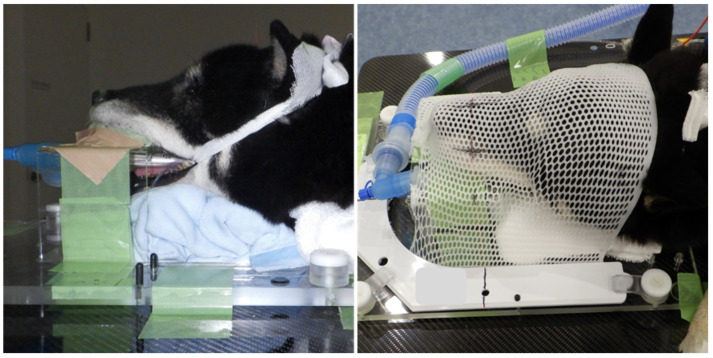
The dog is shown in an immobilization device for imaging and radiotherapy of the nasal tumor.

The patient was positioned in ventral recumbency using immobilization devices, including an inflatable matless (Vac-Lok^TM^ Cushion, Toyo Medic, Tokyo, Japan), a custom-made thermoplastic face mask, a baseplate (Uniframe ^®^, Toyo Medic, Tokyo, Japan), and bite block (Express ^TM^ impression material, 3M Japan, Tokyo, Japan) (Figure 3).

The radiation plan was devised by veterinarians who used planning software for contouring and treatment planning (Monaco, Elekta Japan, Tokyo, Japan) using the Monte Carlo equivalent algorithm. Pre- and post-contrast CT images were co-registered prior to contouring. RT was delivered using 6 MV photons from a linear accelerator (Elekta Synergy, Canon Medical Systems, Otawara, Japan). The linear accelerator was equipped with a 160-leaf, 0.5 cm multileaf collimator, which was used to shape the fields to expose the target volume and shield the adjacent normal tissue. In the first week, the dog received 4.2 Gy per fraction using arc radiotherapy, with four fractions per week for a total of four fractions (total, 16.8 Gy). After the second week, the dog received 2.8 Gy per fraction using a step-and-shoot technique. Four fractions per week made a total of 11 fractions (total, 30.8 Gy).

On follow-up examination 1 month after completing the course of RT, the clinical signs were unchanged, and the nasal tumor’s appearance on CT indicated a mild increase in size. The owner declined further treatment; thus, information regarding further progress was unavailable, as the patient did not return to the clinic.

### 2.2. Thermography and Behavioral Analysis

**Figure 4:**
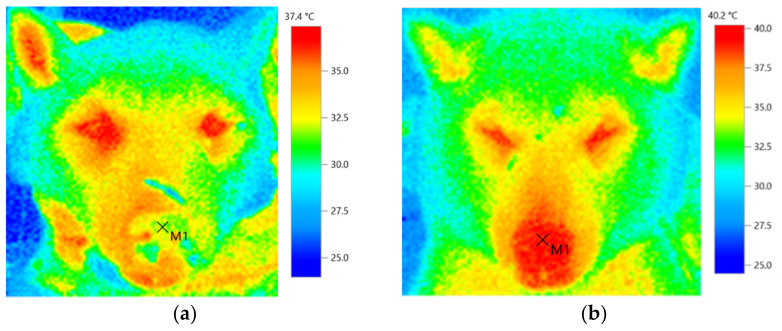
Thermal images before RT ((**a**) before induction of anesthesia for RT) and after RT ((**b**) 210 min after RT). M1 (×) was the target region used for thermographic analysis.

Thermographic photography was performed using an infrared-based Testo 868 thermal imaging camera (Testo SE and Co. KGaA, Yokohama, Japan) with the basic technical parameters: image resolution, 160 × 120 pixels; thermal resolution, <0.1 °C; measuring range, −30 to 650 °C; and accuracy, ±2 °C. Thermal imaging was performed before induction of anesthesia for RT and after RT in a room with controlled room temperature (23–26 °C) and room humidity (50–65%). Although post-RT imaging was performed approximately every hour after irradiation, there were discrepancies in the time due to treatment, examination, and discharge time. Therefore, in this case, the data were analyzed by dividing the time into three blocks: 30–120 min (T1), 121–240 min (T2), and 241–360 min (T3) after RT. The nasal planum in the center of the right and left external nostrils was photographed from the front, while held lightly in a sitting position to prevent the face from moving. To reduce the effects of environmental factors on thermographic readings, all images were scanned at the same distance (1 m) from the patient [22]. The thermal images were analyzed using Testo IR software version 4.7 (Testo SE and Co. KGaA, Yokohama, Japan) (Figure 4a,b).

Behaviors were recorded in the same kennel before and after RT. After acclimation for at least 15 min, a video camera was set up in front of the room containing the patient and filmed for 10 min unattended. Post-RT behaviors were recorded after confirming that the patient was conscious and able to drink independently, taking into account the effects of anesthesia. In this case, the images were taken 3–4 h after RT. The behavioral analysis was based on 13 items: head up, head down, licking, panting, mouth breathing, standing, sitting, yawning, back posture, nausea, sniffing, whining, and stretching (Table 1). We measured the number of seconds each action was performed over 10 min, and the percentage was calculated. The acute pain scale (a common way to assess pain in a veterinary clinic) for dogs developed by the Japanese Society for the Study of Animal Pain was used to assess pain [23] and was evaluated in real time. Body temperature measurements before and after RT were recorded after the video.

### 2.3. Statistical Analyses

All statistical analyses were performed using GraphPad Prism 9.0 (GraphPad Software, Inc., San Diego, CA, USA). The statistical significance of the thermal image data was determined using the Kruskal–Wallis test and Dunn’s multiple comparisons tests. Behavioral and body temperature data were analyzed using the Mann–Whitney U test. Differences were considered statistically significant at *p* < 0.05.

## 3. Results

**Figure 5:**
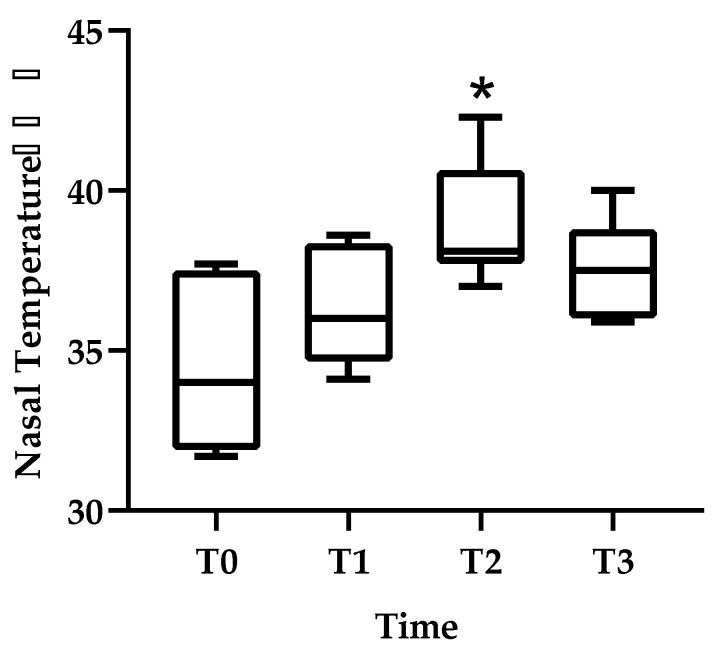
Changes in nasal planum temperature are shown before RT (T0), 30–120 min after RT (T1), 121–240 min after RT (T2), and 241–360 min after RT (T3). Thermal image data are presented as mean ± standard deviation (SD). * Indicates statistical significance (T0 vs. T2).

The nasal planum temperature was (median (range)) 34.0 (31.7–37.7) °C for T0, 36.0 (34.1–38.6) °C for T1, 38.1 (37.0–42.3) °C for T2, and 37.5 (36.0–40.0) °C for T3. There was a significant increase in T2 compared to T0. Compared to pre-irradiation, the nasal planum temperature had increased by 2 °C by T1, 4.1 °C by T2, and 3.5 °C by T3 (Figure 5). Behavioral analyses before and after RT indicated no statistical differences in head up, mouth breathing, or head down percentages (Table 2). No statistically analyzable data were available for licking, standing, sitting, yawning, back posture, nausea, sniffing, whining, and stretching because the patient rarely displayed these behaviors. Post-RT pain levels evaluated by the pain scale ranged from 0 or 1 out of a range of 0 (minimal) to 4 (severe). No painful side effects, such as burns, ulcers, and self-destruction, were observed. Body temperature was similar before RT (median (range)—37.8 (37.5–38.3) °C) and after RT (37.9 (37.6–38.2) °C).

## 4. Discussion

In this report, we evaluated nasal planum temperature and behavioral changes before and after RT and pain levels after RT in a dog with nasal cavity tumors. The nasal planum temperature gradually increased after irradiation and was significantly higher at 120–240 min, and the maximum temperature was 42.3 °C. In addition, the nasal planum temperature increased by more than 2–4 °C after irradiation, and the median temperature was over 36 °C from 2 h after RT.

For healthy dogs, body surface temperature, as measured by thermography, is approximately 30–35 °C [24]. In addition, thermographic readings are lower than rectal temperature [24]. Even in studies investigating body surface temperature after exercise, the increase is only approximately 1 °C [25]. Furthermore, in a report investigating the effects of thermotherapy on arthritis using thermography, the body surface temperature after treatment was approximately 36 °C [26]. Due to the relatively moist nature of dogs’ noses, the area tends to have low temperatures in thermal images [17]. Although there are no reports of a correlation between body surface temperature (measured by thermography) and discomfort index for dogs, it can be assumed that the increase in body surface temperature above 36 °C observed in this case is unlikely to occur in untreated dogs and indicates a higher temperature than it appears.

Reports on humans and rats indicate that temperature changes in the superficial tissues of ≥1 °C reflect inflammation in the subcutaneous and deeper tissues [14,15,16], suggesting that some tissue reactions occurred at the irradiation site in this case. In humans, an increase in skin temperature after radiation causes psychological distress, such as pain and discomfort, which leads to a decrease in the patient’s quality of life [21,22]. According to a report on humans who received RT for breast cancer, the discomfort index increased significantly when the difference in body surface temperature between the unirradiated and irradiated areas exceeded 1 °C, and the body surface temperature after treatment when the discomfort was felt was about 37.4 °C [12]. Hence, no pain or behavioral changes emerged after irradiation in this case, but the temperature of the irradiated area rose by more than 2 °C after irradiation. Additionally, the nose, which is a sensitive area, reached 42.3 °C, which may have caused discomfort for the dog.

In this case, no significant difference was observed in behavioral changes pre- and post-RT, and the pain evaluation was 0 or 1, which did not indicate that treatment was required. Thus, no veterinary treatment was conducted. In veterinary practice, behavioral analysis and pain scales are mainly used to assess the levels of distress and pain in animals. However, stress-related behaviors, such as pain and discomfort, have been reported to vary among individuals, including sex, breed, and past experience [27,28,29]. In addition, because various staff members working with the same patient in an animal hospital, there is a limit to judging dogs’ distress based only on subjective assessment methods, such as appearance and behavioral assessments. Furthermore, unlike surgical procedures, RT is minimally invasive, and it is difficult to observe physical changes after treatment. On the other hand, thermal imaging cameras may be a tool for estimating discomfort, even for patient dogs, because they can objectively evaluate the animal’s condition.

In the present study, the increase in nasal planum temperature was detected 120–240 min after irradiation. This finding indicates that RT may cause discomfort later compared to invasive procedures, including surgery, which may cause pain and discomfort immediately after the procedure. As a result, we may be unwittingly causing animals to overlook their pain and discomfort. Therefore, prolonged observation and care is necessary after RT.

In this case, the highest temperature of the nasal planum was 42.3 °C, which was much higher than the normal body temperature of the dog. When the cellular temperature increases over 42 °C, it causes long-term cell inactivation [27]. Taking advantage of this, hyperthermia, including radiofrequency ablation and hyperthermia, is used to treat cancer [30,31,32]. Therefore, a rise in temperature after RT may be due to a synergistic effect of RT. However, this case did not demonstrate this synergy for RT. Alternatively, since pain and discomfort may occur when the skin temperature rises, future investigation is necessary to assess the need for providing cooling measures against the high temperature after RT.

## 5. Conclusions

This is the first report on a dog that used serial thermographic imaging to analyze the results of radiation therapy to the nasal planum. There were no significant changes in behavior or pain ratings before and after RT, but the nasal planum temperature increased significantly after RT and reached a peak of 42.3 °C at 120–240 min. An increase in skin temperature at the irradiated site can cause tissue reactions that may be uncomfortable for the animal. Therefore, prolonged observation and care are necessary after RT.

A thermographic assessment can be used as a simple, non-invasive way to objectively evaluate changes in an animal’s condition that cannot be observed by general nursing monitoring alone. However, further investigation is needed to determine the correlation of elevated nasal planum temperatures with pain and discomfort. Future prospective studies with more cases will enable detailed evaluations of the state of animals after RT and lead to appropriate nursing care.

## Figures and Tables

**Table 1:** Ethogram for the patient relevant to radiation therapy.

Behavior	Operational Discription
Sniffing	Muzzle/nose oriented in a clearly observable direction
Licking	Licking nose or lip
Yawning	Opening mouth widely and inhaling
Panting	Tongue exposed and observable breathing
Mouth breathing	Observable breathing through the mouth
Stretching	Extending body and one or more front and/or hind legs while remaining stationary
Whining	A cyclic vocalization
Nausea	Abnormal heaving or chewing
Head down	Lying down with head placed on front paws
Head up	Lying down with head away from front paws
Standing	Supported upright with all four legs
Sitting	Supported by two extended front legs and two flexed back legs
Back posture	Showing back against the camera

**Table 2:** Behavioral changes before and after RT.

	Before RT [Median (Range)]	After RT [Median (Range)]	*p*-Value
Head up	27.8 (0–49.4)	20.4 (2.8–40.6)	0.53
Mouth breathing	3.4 (0–13.4)	2.6 (0–18.3)	0.98
Head down	14.3 (0–36.8)	29.3 (0–46.9)	0.17

## Data Availability

Not applicable.
